# Thiourea improves yield and quality traits of *Brassica napus* L. by upregulating the antioxidant defense system under high temperature stress

**DOI:** 10.1038/s41598-024-62257-y

**Published:** 2024-05-28

**Authors:** Muhammad Ahmad, Ejaz Ahmad Waraich, Usman Zulfiqar, Jean Wan Hong Yong, Muhammad Ishfaq, Kaleem ul din, Aman Ullah, Adeel Abbas, Masood Iqbal Awan, Ihab Mohamed Moussa, Mohamed S. Elshikh

**Affiliations:** 1 Department of Agronomy, University of Agriculture, Faisalabad, 38040 Pakistan; 2 Department of Agronomy, Faculty of Agriculture and Environment, The Islamia University of Bahawalpur, Bahawalpur, 63100 Pakistan; 3 Department of Biosystems and Technology, Swedish University of Agricultural Sciences, Alnarp, Sweden; 4 Department of Agriculture, Extension, Azad Jammu & Kashmir, Pakistan; 5 Department of Botany, University of Agriculture, Faisalabad, 38040 Pakistan; 6 Institute of Environment and Ecology, School of Environment and Safety Engineering, Jiangsu University, Zhenjiang, 212013 People’s Republic of China; 7 Department of Agronomy, University of Agriculture, Faisalabad, Depalpur-Okara Campus, Pakistan; 8 Department of Botany and Microbiology, College of Science, King Saud University, 11451 Riyadh, Saudi Arabia

**Keywords:** Antioxidants, Canola genotypes, Electrolyte leakage, Fatty acid profile, Hydrogen peroxide, Lipid peroxidation, Plant sciences, Plant physiology

## Abstract

High temperature stress influences plant growth, seed yield, and fatty acid contents by causing oxidative damage. This study investigated the potential of thiourea (TU) to mitigate oxidative stress and restoring seed oil content and quality in canola. The study thoroughly examined three main factors: (i) growth conditions—control and high temperature stress (35 °C); (ii) TU supplementation (1000 mg/L)—including variations like having no TU, water application at the seedling stage, TU application at seedling stage (BBCH Scale-39), water spray at anthesis stage, and TU application at anthesis stage (BBCH Scale-60); (iii) and two canola genotypes, 45S42 and Hiola-401, were studied separately. High temperature stress reduced growth and tissue water content, as plant height and relative water contents were decreased by 26 and 36% in 45S42 and 27 and 42% Hiola-401, respectively, resulting in a substantial decrease in seed yield per plant by 36 and 38% in 45S42 and Hiola-401. Seed oil content and quality parameters were also negatively affected by high temperature stress as seed oil content was reduced by 32 and 35% in 45S42 and Hiola-401. High-temperature stress increased the plant stress indicators like malondialdehyde, H_2_O_2_ content, and electrolyte leakage; these indicators were increased in both canola genotypes as compared to control. Interestingly, TU supplementation restored plant performance, enhancing height, relative water content, foliar chlorophyll (SPAD value), and seed yield per plant by 21, 15, 30, and 28% in 45S42; 19, 13, 26, and 21% in Hiola-401, respectively, under high temperature stress as compared to control. In addition, seed quality, seed oil content, linoleic acid, and linolenic acid were improved by 16, 14, and 22% in 45S42, and 16, 11, and 23% in Hiola-401, as compared to control. The most significant improvements in canola seed yield per plant were observed when TU was applied at the anthesis stage. Additionally, the research highlighted that canola genotype 45S42 responded better to TU applications and exhibited greater resilience against high temperature stress compared to genotype Hiola-401. This interesting study revealed that TU supplementation, particularly at the anthesis stage, improved high temperature stress tolerance, seed oil content, and fatty acid profile in two canola genotypes.

## Introduction

Besides the fundamental importance of plant species genotypic variability, abiotic stresses are the other major limiting factors constraining plant growth and development^1–3^. Thermal stress, or exposing plants to unfavourable growth temperatures, is a major inhibitor of plant metabolism that affects crop production systems globally^2,5,6^. Climate change is rapidly becoming a serious and more frequent disruptor to the world's food security due to ever-increasing atmospheric temperature increases causing global warming^7^ as each degree rise in global temperature would decrease crop yield by 17%^8^ and 10–20%^9^. Through a major meta-analysis conducted through different models, it was predicted that there would be higher heat wave frequency leading to warm days and nights; thereby increasing global temperatures from 2.0 to 4.9 °C at the end of the twenty-first century^10^.

Canola (*Brassica napus* L.) belongs to Brassicaceae known as the double-low and double-zero crop due to the low glucosinolate and low erucic acid contents in seeds^1^. Canola is an important winter oilseed crop that has a major role in world oilseed production, and it has gained greater recognition in the recent past^11,12^. Being a cool-season crop, canola has a greater sensitivity towards heat stress^13^ particularly at anthesis^14,15^. The mean air temperature may increase up to 27 °C even in the cooler regions at the fluorescence initiation stage which may lead to a disturbed fertilization process and yield losses in canola,^15^ as yield contributing parameters, including, number of seeds, weight of seeds, and seed yield, and may be adversely affected by the elevated temperature stress^16,17^. Under high temperature stress, the most damaging mechanism of heat injury is the excessive production of reactive oxygen species (ROS) damaging cell structure, membrane permeability, and impaired plant metabolism^18,19^. ROS activity induces H_2_O_2_ content that leads to impaired antioxidant defense that becomes highly hazardous for cellular redox due to its high oxidizing powers^20,21^. High temperature stress has several effects on canola productivity particularly at the reproductive stage. When canola is developing reproductively, high temperature stress may affect flower initiation, pollination, fertilization, and oil content^22^. For canola, the two most important economic criteria are seed yield and seed oil contents. As a result, the worldwide oilseed trade will be impacted if high temperature stress causes a decline in seed oil content and fatty acid content^23^. The degree, timing, and length of exposure to high temperature stress also determine how sensitive sexual reproduction is to high temperatures. Even a short period of high temperature stress at flowering can have a negative impact on seed quality parameters. However, the heat sensitivity of canola has been reported at 29.5 °C, 30 °C, 35 °C, and 31 °C^24–26^. These studies have reported a yield loss in canola due to high temperature stress at sensitive crop stages causing a decrease of 15–20%^27^, 55%^26^, and 38%^1^. High temperature stress generally affects the quantity and quality of canola^28^.

To mitigate heat-induced damage, plants will up-regulate various ROS scavenging mechanisms by activating plant defense systems depending upon enzymatic and nonenzymatic antioxidants^27,29,30^. Plants may adopt high temperature stress through natural mechanisms, however, different biochemical compounds called plant growth regulators (PGRs), upregulate antioxidant defense systems to improve crop resilience under high temperature stress^31^. An array of synthetic compounds is known for their growth-regulatory properties, while thiourea (TU) is a biological molecule that has an important role in resilient crop production and works as a source of nitrogen nutrition^32^. Thiourea, a substance that controls plant growth, is a powerful hydroxyl compound that detoxifies the ROS that guards’ plants against the oxidative damage that high temperature stress can cause while also regulating the cascades of plant phenological attributes^33,34^. Thiourea has two important functional groups that make it an important stress-alleviating molecule ‘thiol (SH)’, which has a role in alleviating negative effects of high temperature, and “imino (NH_2_)”, which has a role in plant nutrition under stressed conditions^35^. As a stress-alleviating compound, TU can maintain plant redox potential imparted by –SH group^33^ and improve the plant metabolism for successful crop production under unfavorable environments^28,36^. Nitrogen can improve plant growth and development by improving the chlorophyll content in plants ultimately improving the photosynthetic rates in plants^35,37^. Thiourea applications certainly improve the heat shock proteins (HSPs) in plants to impart high temperature stress tolerance^38^. The role of TU has been documented to alleviate abiotic stresses including drought, salt stress, heavy metal stress, and high temperature stress^39^. Thiourea applied in plants improves their physicochemical attributes to support plant growth^40^. Additionally, in the current scenario, research tends to focus on locating and utilizing TU molecules to mediate high temperature stress tolerance of canola genotypes. Thus, the goal of this study was to explore the potential of the environmentally friendly substance TU, which has been used to reduce oxidative stress in canola.

Thiourea applications to improve plant growth and development have been documented^1,31, 34,36^. However, the role of TU to improve seed oil content and oil quality parameters needs to be investigated under unfavorable environmental conditions. The study hypothesized that TU supplementation alleviates the oxidative stress caused by high temperature stress and restoring the canola seed oil and oil quality (unsaturated fatty acids) in due process. The objectives of the study were, i) to evaluate the role of TU in conferring high temperature stress tolerance in canola genotypes, and ii) to assess the role of TU application on seed oil content and fatty acid profile in canola genotypes.

## Materials and Methods

### Experimental treatments

The study was conducted in sandy soil in plastic pots (38 cm and 24 cm). The TU dose (1000 mg/L) was pre-optimized and used in this study^1^. Two canola varieties (G_1_ = 45S42 (heat tolerant) and G_2_ = Hiola-401 (heat sensitive)^1^ was used independently in this study, the seeds were obtained from, Pioneer (45S42), and Imperial Chemical Industries (ICI) (Hiola-401), Pakistan. The genotypes were selected as one sensitive and one tolerant genotype from a preliminary experiment where we used 4 genotypes including Faisal canola, Super canola, Hiola-401, and 45S42. In the end, we selected the most tolerant and most sensitive genotype based on the results of physiological indices (data not published). Results showed that 45S42 was the most tolerant genotype and Hiola-401 was the most sensitive genotype which was tested in a preliminary study to optimize TU rates^1^. The experiment was laid out in a completely randomized design (CRD) with the factorial arrangement and three replications. The study comprised of two factors including (i) Growth conditions; NS = control-no stress (25/18 °C day/night) and HT = high temperature stress (35/25 °C day/night), (ii) TU supplementation; NA = control-no TU supplementation, WA_1_ = water spray at seedling stage (Principal growth stage 3: Stem elongation, BBCH Scale-39), 39 days after sowing (DAS), TU_1_ = foliage TU supplementation at seedling stage (Principal growth stage 3: Stem elongation, BBCH Scale-39), 39 DAS, WA_2_ = water spray at anthesis stage (Principal growth stage 6: Flowering, BBCH Scale-60), 60 DAS, and TU_2_ = foliage TU supplementation at anthesis stage (Principal growth stage 6: Flowering, BBCH Scale-60), 60 DAS^40,41^. Thiourea (98% purity; Sigma-Aldrich), with a molecular weight of 76.12 g/mol, is also known by the alternate name thiocarbamide, according to the International Union of Pure and Applied Chemistry (IUPAC). To create the thiourea molecule, sulphur was added in place of oxygen in the urea molecule. Because of this substitution, thiourea's characteristics differed significantly from those of urea. At 25 °C, it dissolves in water sparingly, with a solubility of 142 g/L^1,28^. The experiment comprised 60 pots, in two sets, each containing 30 pots; set-1 (45S42) and set-2 (Hiola-401). Following which, these two sets were further divided into 2 sets, the control containing 15 pots and the high temperature stress treatment consisting of other 15 pots.

The basic crop nutrition was delivered by applying modified Hoagland’s solution (6 mM KNO_3_; Ca (NO_3_)_2_· 4 mM 4H_2_O; 1 mM NH_4_H_2_PO_4_; 46.2 μM H_3_BO_3_; 2 mM MgSO_4_·7H_2_O; 9.1 μM MnCl_2_·4H_2_O; 0.3 μM CuSO_4_·5H_2_O; 0.8 μM ZnSO_4_·7H_2_O; 0.1 mM Fe–Na_2_–EDTA)^18^. The plastic pots (Length = 38 cm and Width = 30 cm) filled with 10 kg of sandy loam soil, were used for the seed sowing. Initially, ten seeds of both canola genotypes were sowed in each replicated pot. After emergence, only six plants/pots were kept for the data collection. Plants were raised at a normal temperature of 25/18 °C Day/night in two sets in two different growth rooms having the same internal conditions till anthesis (60 DAS), whereas in one growth room heat treatments were applied at 35/25 °C by maintaining the moisture level in each pot, 60% humidity, and 8 h light, 2 °C temperature increased on daily basis till it reached 35/25 °C. The internal conditions were controlled by the mechanized units of cooling, high temperature, humidifier/dehumidifier adjustment systems, and light (∼12,000 lx). The temperature around crop canopies was recorded by digital maximum and minimum thermometers (Youshiko-YC9070, Japan). The pot moisture content was maintained by adding water on a weight basis by using digital balance (TX323L, Shimadzu, Japan), as the number of grams decreased in pot weight was brought to the original weight (field capacity) by adding water.

### Measurements

#### *Morpho*-physiological parameters

The measurement of growth parameters was done 72 DAS (Principal growth stage 7: Development of fruit)^40,41^, including A total of 120 plants, including two from each replication, which were used to measure root length, plant height, root fresh weight, shoot fresh weight, root dry weight, and shoot dry weight. A meter rod was used to measure the height of the plant from the soil's surface to its tip and averaged, while root length was also measured with the meter rod. The root and shoot fresh weight were recorded by using a digital weighing balance (TX323L, Shimadzu, Japan). Dry weight was recorded with an oven (Memmert-110, Schawabach, Germany) at 70 °C for 48 h.

The leaf water relations were measured two days after stress implications at 72 DAS (Principal growth stage 7: Development of fruit)^40,41^. The youngest top third fully expanded leaf was harvested from each treatment to measure leaf water potential (Ψw). The data was recorded with a Scholander type pressure-chamber (ARIMAD-2, ELE-International, Japan) from 8:00 a.m. to 10:00 a.m. to measure water potential. These leaves were in a freezer at -20 °C to determine osmotic potential (Ψs). By melting and crushing the frozen leaves with a glass rod the cell sap was extracted with a disposable syringe. Subsequently, the sap was placed in an osmometer (Wescor-5500, USA) to measure the osmotic potential, and the pressure potential (Ψp) was obtained by using the formulae;

$$(\Psi {\text{p) = (}}\Psi {\text{w) - (}}\Psi {\text{s)}}$$

To measure the leaf relative water content (RWC), the samples were taken at 72 DAS (Principal growth stage 7: Development of fruit)^40,41^. The RWC was recorded following the process of Mullan and Pietragalla^42^.

The top third-greenest leaf at 72 DAS (Principal growth stage 7: Development of fruit)^40,41^ was measured for chlorophyll intensity using a Soil Plant Analysis Development (SPAD) chlorophyll meter (SPAD-502 Plus; Konica Minolta, Osaka, Japan). SPAD values for each pot were recorded and averaged^43^.

#### Yield and Yield quality parameters

To determine seed yield and yield attributes, the harvesting was done at 121 DAS at maturity (Harvesting stage). Hundred seeds were manually counted and data was recorded by a digital balance (TX323L, Shimadzu, Japan). All the pods were manually harvested to determine the seed yield/plant.

Utilizing near-infrared reflectance spectroscopy to determine the seed oil and protein contents (on a dry-matter basis) (NIRS-Model-6500), canola seed samples from each replication were obtained on a dry-matter basis^44^.

Anderson et al.^45^ described the process to measure fatty acid content. By employing a gas chromatograph (Trace-1310, Thermo-Scientific, Waltham, MA) equipped with an auto-sampler, a flame-ionization detector, and fatty acids in canola oil were expressed as percentages and quantified. The analyses were performed by a DB-23 capillary column of 30 m × 0.25 mm (Agilent Technologies, Inc., Santa-Clara, CA) by using detector-temperature, injector, and maintained the flow of He gas (200 kPa at 1.9 mL min^−1^) (230 and 250 °C), respectively. The temperature rose from 190 °C for 4 min to 220 °C for 15 min before remaining at 220 °C for 1 min. The standards utilized as references are 17A, 21A, 68B, and 411 (Nu-Chek-Prep, Inc., Elysian, MN), which contain a variety of fatty acid (FA) methyl esters. The standards' retention periods for each sample were used to identify the peaks, and Chromeleon v7.2 software was used to measure the peak-area (Thermo-Scientific).

#### Biochemical parameters

The sample leaves were harvested with a scissor at 73 DAS (Principal growth stage 7: Development of fruit)^40,41^ to determine superoxide dismutase (SOD), peroxidase (POD), and catalase (CAT). The process of Dhindsa et al.^46^ was followed to determine the activity of SOD; with further modification from Wu et al.^47^ Leaf samples of 200 mg were homogenized in phosphate buffer (2 mL, pH 7.5, 0.1 M) + EDTA (0.5 mM) centrifuged at 10,000 rpm and stored at 4 °C. The same supernatant was used to test SOD activity based on its capacity to prevent the photochemical reduction of nitro-blue tetrazolium. For 15 min, a 3 mL assay mixture containing two 15 W fluorescent lights was incubated, with lit and unilluminated reactions serving as calibration. A spectrophotometer (UV-4000, ORI, Germany) was used to measure the absorbance of the supernatant with a blank wavelength of 560 nm. At 4 °C the homogenates were centrifuged for 20 min at 15000 rpm and the activities of CAT and POD were determined from the research mixture. The method used by Liu et al.^48^ to assess CAT activity was used. Freshly primed-hydrogen peroxide (5.9 mM, 35% pure, 100 µL) and enzyme extract (100 µL) were mixed to start the process. The disappearance rate of H_2_O_2_ at 240 nm was measured using a microplate reader (Bio-Tek Instruments, ELX800, Inc., Winooski, VT, USA) that observed the decrease in absorbance for three minutes at 240 nm.

POD activity was determined with slight modifications in the procedure proposed by Putter,^49^. The supernatant having 5 mM of H_2_O_2_, 10 mM of guaiacol, and 50 mM phosphate buffer (pH 7.0) was preheated in a water bath at 20 °C. Then, 2.8 mL of reaction solution and 0.2 mL enzyme were added in a 10 mL centrifuged tube and thoroughly mixed. The absorbance of the supernatant was measured with a spectrophotometer (UV-4000, ORI, Germany) for one minute.

The osmolytes were determined at 73 DAS (Principal growth stage 7: Development of fruit)^40,41^ to assess proline content^50^ and glycine betaine^51^. The samples were grounded and vigorously shaken at 25 °C with 20 mL deionized water and after filtration 2N H_2_SO_4_ was added to dilute a solution and cortex mixture was stirred with cold reagent (KI-I2). Test tubes containing the reagents were centrifuged for 15 min at 10,000 rpm after being kept at 4 °C for 16 h. The absorbance of supernatant was measured using a spectrophotometer (UV-4000, ORI, Germany) at a wavelength of 365 nm. Using ninhydrin, the amount of free proline in the leaves was determined. Fresh leaf samples were homogenized and pulverized in 3 mL of ice-cold 5-sulfosalicylic acid using a chilled mortar and pestle. In glass test tubes, plant extracts (2 mL) were combined with glacial acetic acid (2 mL), and acid ninhydrin (2 mL), and incubated for 1 h in a hot water bath. The reaction was then finished in an ice bath. Toluene (4 mL) was added to the reaction mixture in the test tubes and shaken vigorously for 20–25 s. At 520 nm, spectrophotometric absorbance was measured and GB was determined.

The sampling was done at 73 DAS (Principal growth stage 7: Development of fruit)^40,41^ to determine total soluble proteins^52^ and total soluble sugar content^53^. One milliliter of the leaf extract was put into test tubes. One mL of phosphate buffer was present in the blank (pH 7.0). Alkaline solution (1 mL) was added to a test tube along with other ingredients, and it was let to stand for 10 min at room temperature. Then, 0.5 mL of diluted (1:1) and thoroughly mixed Folin-Phenol reagent was incubated at room temperature for 30 min. Optical density was measured by using a spectrophotometer at 620 nm. The plant material (0.1 g) was extracted for soluble sugars in an ethanol solution (80%), which was then incubated at 60 °C for 6 h, and Optical density was measured by using a spectrophotometer at 625 nm.

**Figure 1:**
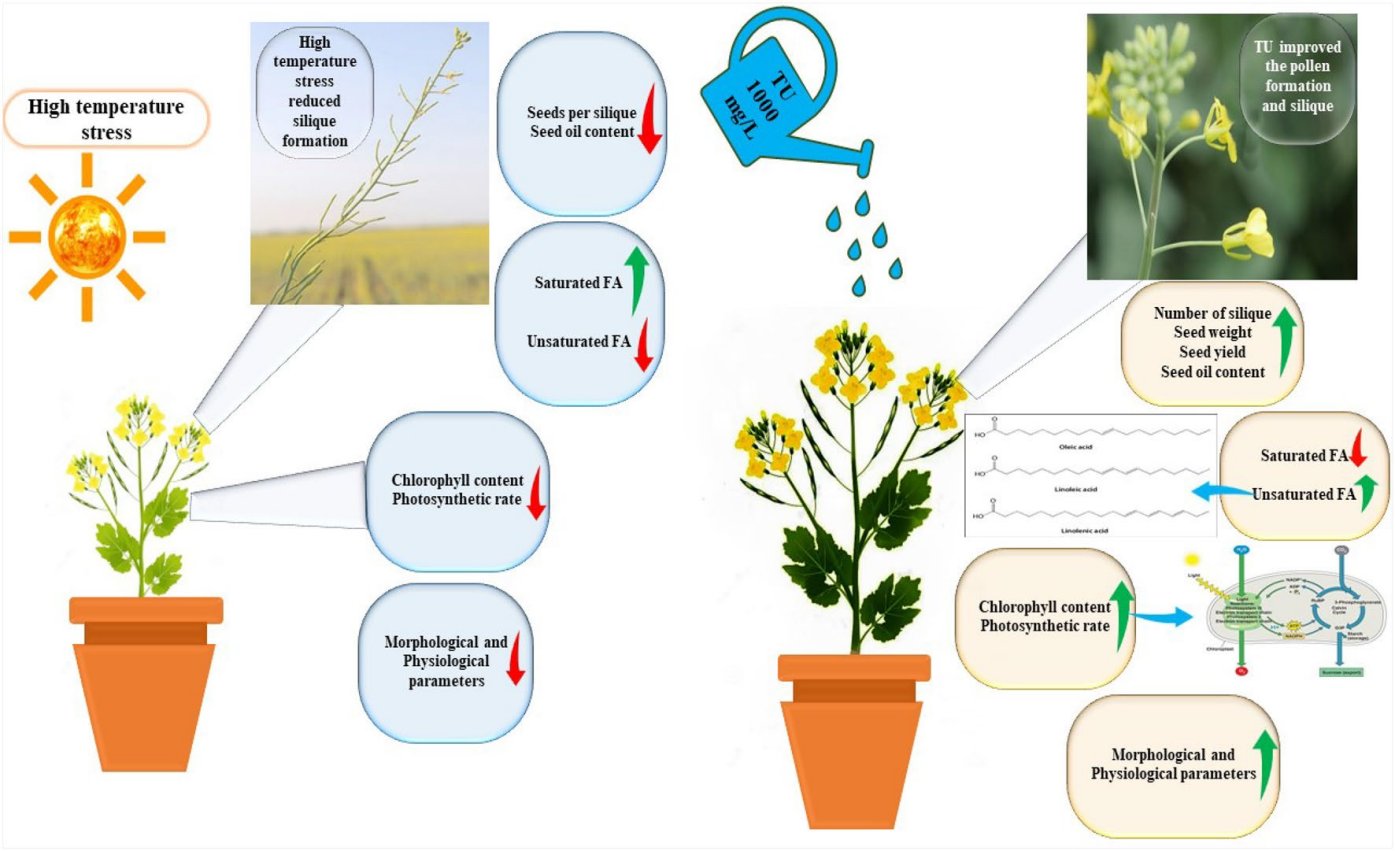
Thiourea applications (1000 mg/L) restored the plant physicochemical attributes, seed yield, seed oil content, and fatty acid profile in canola grown under heat stress (35 °C).

Oxidative analysis was done at 73 DAS (Principal growth stage 7: Development of fruit)^40,41^ according to the methods proposed to measure leaf malondialdehyde (MDA) contents^54^, hydrogen peroxide (H_2_O_2_)^55^, and electrolyte leakage (EL)^56^. The thiobarbituric acid response revealed the extent of lipid peroxidation. After being homogenized in 0.1 percent trichloroacetic acid (1:10, w: v) with frozen samples, centrifuged for 15 min at 10,000 rpm. The supernatant (1 mL) was incubated with 0.5% thiobarbituric acid (4 ml) in trichloroacetic acid (20%) for 30 min at 95 °C in a fume hood and cooled with ice-bath. The thiobarbituric acid response revealed the extent of lipid peroxidation. After being homogenized in 0.1%trichloroacetic acid (1:10, w: v) with frozen samples, centrifuged for 15 min at 10,000 rpm. Malondialdehyde equivalent was determined for the thiobarbituric acid reactive material concentration using the extinction coefficient (155 mM^-1^ cm^-1^). To measure (H_2_O_2_) level, fresh samples (500 mg) were homogenized with trichloroacetic acid (5 mL) and centrifuged at 12,000 rpm for 15 min. The reaction mixture (0.5 mL) was mixed in potassium iodide (1 mL) + phosphate buffer -0.05 M (0.5 mL, pH 7.0), and a spectrophotometer was used to measure absorbance at 390 nm by using water as blank. Cell membrane permeability was measured by taking a test tube filled with a known volume of distilled water, and leaf samples were vertically placed in them, and heated for 2 h at 32 °C to note the electrical conductivity (EC_1_). The samples were heated once more for 20 min at 121 °C, and this time, EC was recorded as EC_2_ (Fig. 1). The formula used to determine EL's final value:

$$EL = \frac{{EC_{1} }}{{EC_{2} }} \times 100$$

### Statistical analysis

Statistix 10.0 (Analytical Software, Statistix; Tallahassee, FL, USA, 1985–2003) was used to statistically analyze the data using the analysis of variance technique, and treatment means were compared at (*P* ≤ 0.05) by utilizing the least significant difference (LSD)^57^ Graphical representation was done with Sigma Plot 10.0, and the correlation matrix of data was done with Statistix 10. A polar heat-map with a dendrogram was made by using OriginPro-2022 and supplemented with various analyses^4,58^.

### Plant guidelines

All the experiments were done in compliance with relevant institutional, national, and international guidelines and legislation. High research standards were maintained throughout the experiments and following the various established scientific protocols^58–61^.

### Ethical approval

The authors declare that all the permissions or licenses were obtained to collect the data and that all study complies with relevant institutional, national, and international guidelines and legislation for research ethics.

## Results

**Figure 2:**
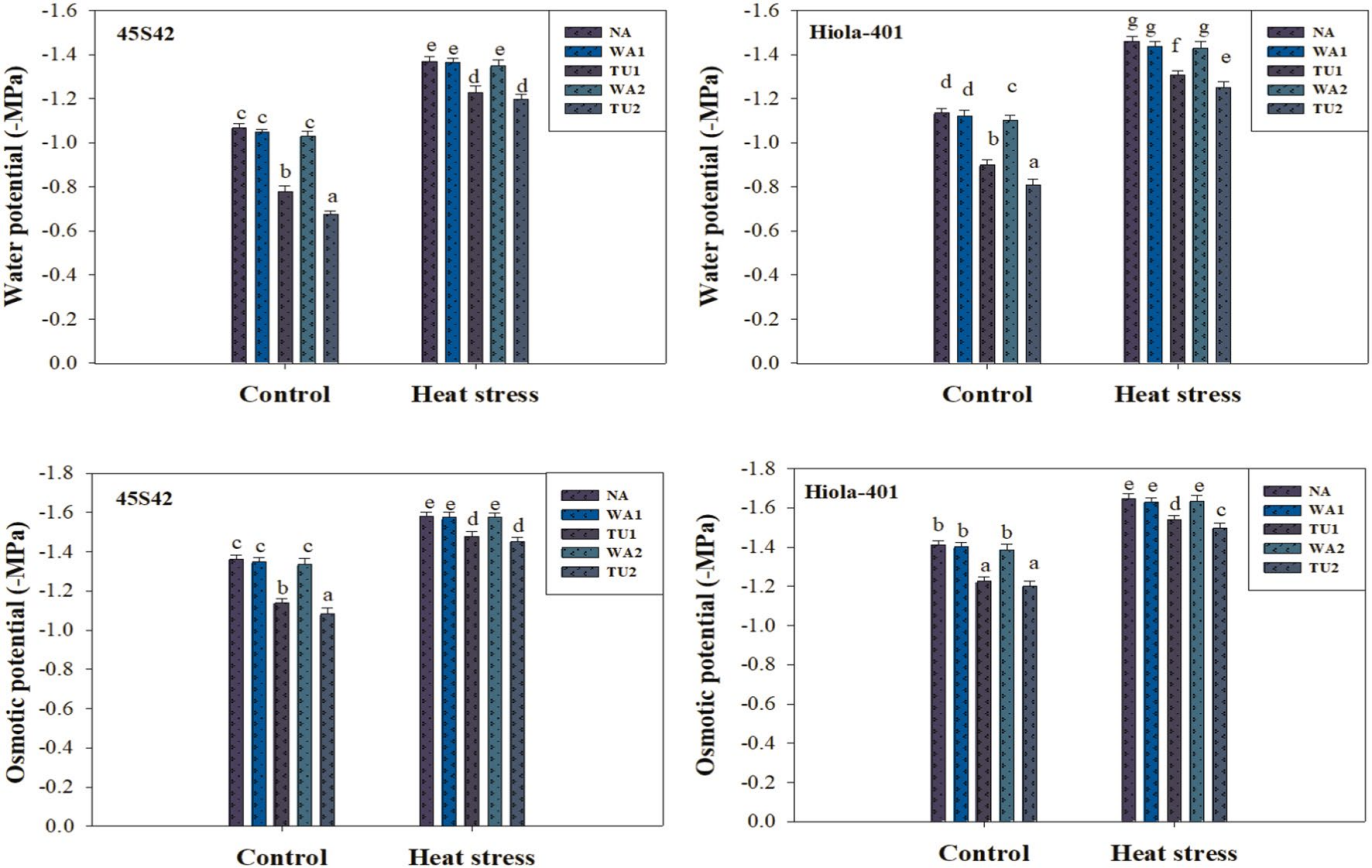
Effects of thiourea applications (NA = Control-no thiourea supplementation, WA_1_ = Water spray at seedling stage, TU_1_ = Thiourea supplementation at seedling stage, WA_2_ = Water spray at anthesis stage, TU_2_ = Thiourea supplementation at anthesis stage) on water potential (-MPa) and osmotic potential (-MPa) in canola genotypes under high temperature stress conditions.

**Figure 3:**
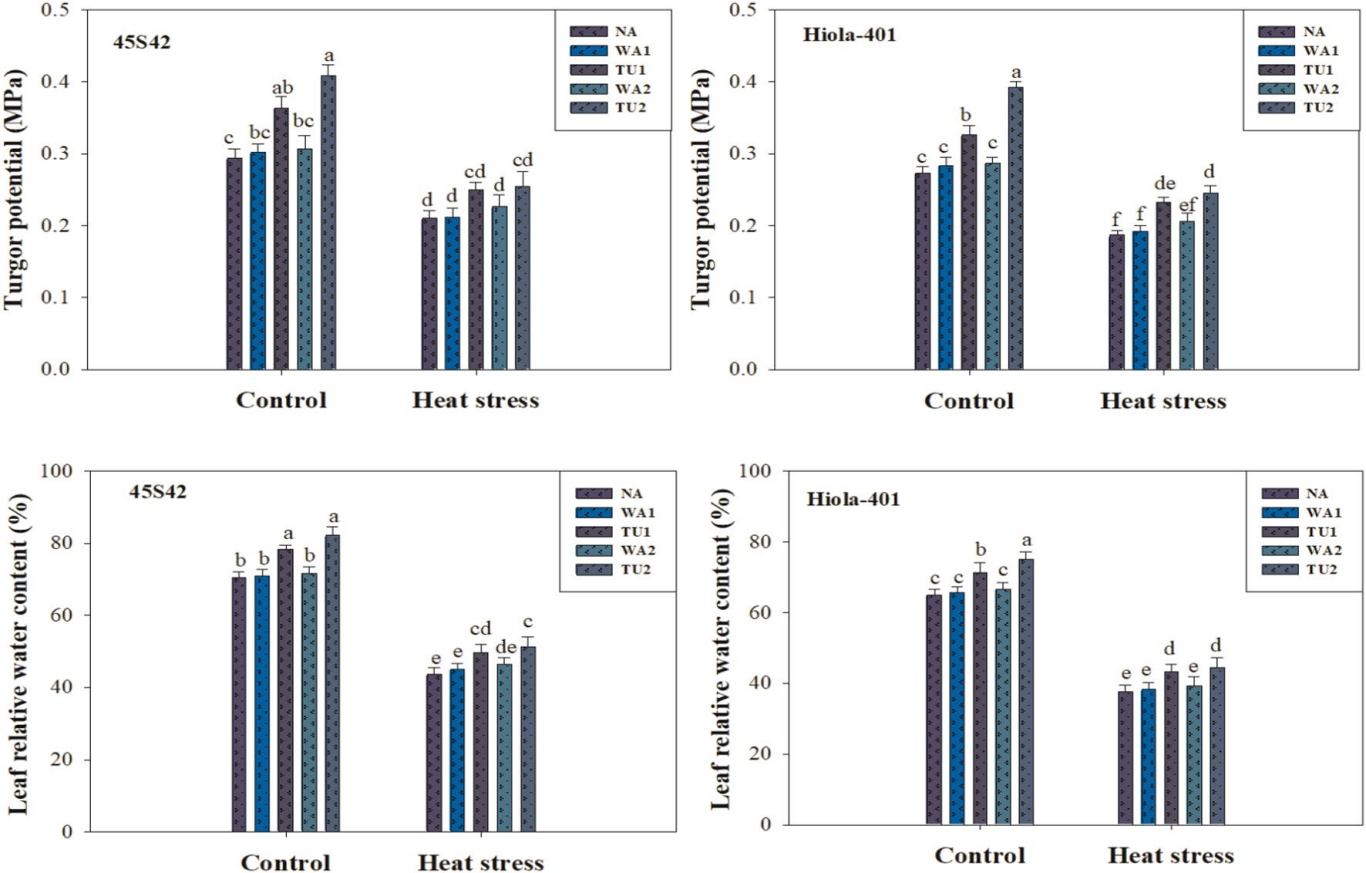
Effects of thiourea applications (NA = Control-no thiourea supplementation, WA_1_ = Water spray at seedling stage, TU_1_ = Thiourea supplementation at seedling stage, WA_2_ = Water spray at anthesis stage, TU_2_ = Thiourea supplementation at anthesis stage) on pressure potential (MPa) and relative water content (%) in canola genotypes under high temperature stress conditions.

**Figure 4:**
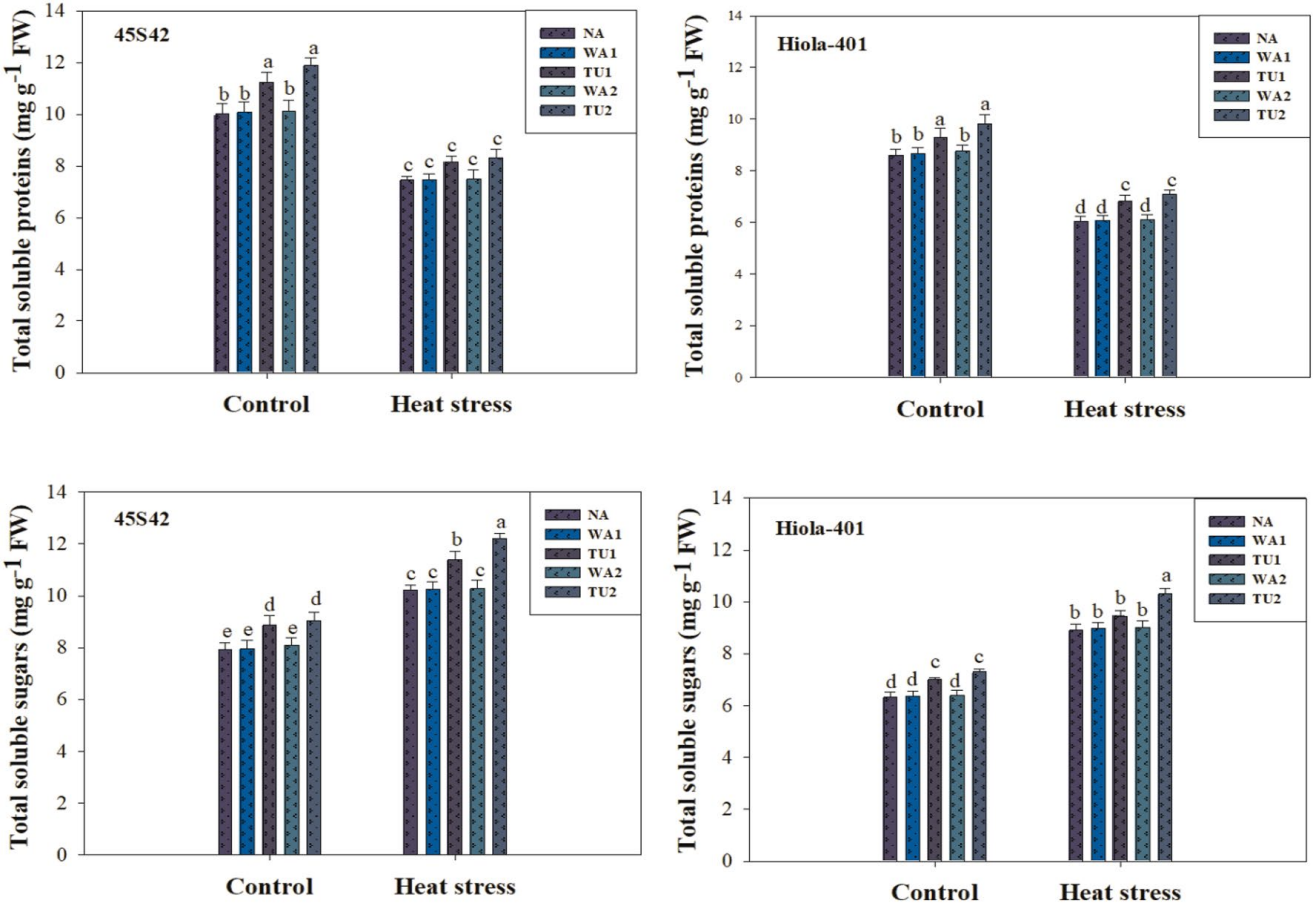
Effects of thiourea applications (NA = Control-no thiourea supplementation, WA_1_ = Water spray at seedling stage, TU_1_ = Thiourea supplementation at seedling stage, WA_2_ = Water spray at anthesis stage, TU_2_ = Thiourea supplementation at anthesis stage) on total soluble proteins (mg g^−1^ FW) and sugars (mg g^−1^ FW) in canola genotypes under high temperature stress conditions.

Results revealed that all the growth, yield, and physicochemical attributes were significantly (*P* ≤ 0.05) affected by high temperature stress and TU supplementation (Tables 1 and 2; Figs. 2–4).

**Table 1:** Effects of supplemental thiourea applications on growth and yield attributes, and foliar chlorophyll (SPAD value) of canola genotypes grown under high temperature stress**.**

Stress conditions (S)	Thiourea application (TU)	Root length (cm)		Plant height (cm)		Dry Weight/plant (g)		SPAD value		100-seed weight (g)		Grain yield/plant (g)	
		G_1_	G_2_	G_1_	G_2_	G_1_	G_2_	G_1_	G_2_	G_1_	G_2_	**G**_**1**_	**G**_**2**_
NS	NA	25.0b	20.0bc	54.3c	48.3c	2.43b	2.33c	2.43b	2.33c	0.24b	0.23c	4.70c	4.52c
	WA_1_	25.6b	21.0bc	54.6c	49.3c	2.44b	2.35c	2.44b	2.35c	0.24b	0.23c	4.74c	4.58c
	TU_1_	31.3a	25.0a	62.6b	56.3b	2.60a	2.44b	2.60a	2.44b	0.26a	0.24b	5.64b	5.14b
	WA_2_	26.6b	22.0b	55.6c	50.0c	2.45b	2.35c	2.45b	2.35c	0.24b	0.23c	4.84c	4.64c
	TU_2_	33.3a	26.6a	70.0a	60.3a	2.62a	2.50a	2.62a	2.50a	0.26a	0.25a	6.27a	5.66a
HT	NA	19.6c	16.0d	40.0e	35.3f.	2.1d	2.04e	2.1d	2.04e	0.21d	0.20e	2.97e	2.77f.
	WA_1_	20.0c	16.3d	41.0e	36.6ef	2.14d	2.05e	2.14d	2.05e	0.21d	0.20e	3.01e	2.81f.
	TU_1_	25.0b	19.3c	47.0d	40.0de	2.28c	2.19d	2.28c	2.19d	0.22c	0.21d	3.73d	3.29e
	WA_2_	20.3c	16.6d	41.6e	37.6ef	2.16d	2.07e	2.16d	2.07e	0.21d	0.20e	3.06e	2.85f.
	TU_2_	26.8b	20.4bc	48.6cd	43.0d	2.34c	2.22d	2.34c	2.22d	0.23c	0.22d	3.95d	3.53d

LSD Least significant difference; Values sharing same case letter or without lettering, for a parameter, do not differ significantly (*P*** ≤ **0.05) by the LSD test.

*’**’***significant at *P* ≤ at 0.05, *P* ≤ at 0.01, *P* ≤ at 0.001 respectively.

NS no stress, HT High temperature stress, NA Control-no thiourea supplementation, WA_1_ = Water spray at seedling stage, TU_1_ = Thiourea supplementation at seedling stage, WA_2_ = Water spray at anthesis stage, TU_2_ = Thiourea supplementation at anthesis stage, G_1_ = 45S42, G_2_ = Hiola-40, ns = Non-significant

**Table 2:** Effects of supplemental thiourea applications on osmolytes, antioxidant enzymes, and oxidative stress of two canola genotypes grown under high temperature stress**.**

Stress conditions (S)	Thiourea application (TU)	Catalase (Unit g^-1^ FW)		Peroxidase (Unit g^−1^ FW)		Superoxide dismutase (Unit g^−1^ FW)		Hydrogen peroxide (μmol g^−1^ FW)		Electrolyte leakage (%)		Malondialdehyde content (μmol g^−1^ FW)	
		G_1_	G_2_	G_1_	G_2_	G_1_	G_2_	G_1_	G_2_	G_1_	G_2_	G_1_	G_2_
NS	NA	47.7e	41.8e	49.9f.	43.1f.	56.5f.	48.3e	0.50d	0.59d	29.5e	33.6d	0.46d	0.55e
	WA_1_	48.5e	42.3e	50.8f.	44.1f.	57.0f.	49.3e	0.49d	0.57d	28.5e	32.7de	0.44d	0.53e
	TU_1_	57.0d	50.9d	64.3de	53.3e	69.9de	59.8d	0.42e	0.50e	24.1f.	27.5f.	0.38d	0.48e
	WA_2_	48.9e	44.0e	51.3ef	45.0f.	57.6f.	51.1e	0.47d	0.56d	27.2e	31.1e	0.42d	0.51e
	TU_2_	59.9d	54.2d	68.2d	57.9d	66.6e	63.7cd	0.37e	0.47e	21.5g	24.3g	0.34d	0.43e
HT	NA	78.0c	67.0c	79.3c	67.1c	74.4cd	63.2cd	1.75a	1.95a	56.3a	63.0a	9.01a	12.0a
	WA_1_	78.3c	68.6c	80.1c	68.7c	75.5cd	65.0c	1.73a	1.94a	54.6ab	61.9a	8.96a	11.8ab
	TU_1_	86.5b	77.0b	95.8b	80.6b	89.1b	75.1b	1.63b	1.84b	47.1c	54.6b	7.72b	10.1c
	WA_2_	79.0c	69.5c	81.2c	70.3c	76.3c	67.0c	1.72a	1.93a	53.3b	61.2a	8.91a	11.5a
	TU_2_	91.8a	83.1a	98.9a	85.1a	95.4a	81.1a	1.55c	1.75c	44.0d	51.7c	6.87c	9.37d

LSD Least significant difference; Values sharing same case letter or without lettering, for a parameter, do not differ significantly (*P*** ≤ **0.05) by the LSD test.

*’**’***significant at *P* ≤ at 0.05, *P* ≤ at 0.01, *P* ≤ at 0.001 respectively.

NS no stress, HT High temperature stress, NA Control-no thiourea supplementation, WA_1_ = Water spray at seedling stage, TU_1_ = Thiourea supplementation at seedling stage, WA_2_ = Water spray at anthesis stage, TU_2_ = Thiourea supplementation at anthesis stage, G_1_ = 45S42, G_2_ = Hiola-40, ns = Non-significant

### *Morpho*-physiological parameters

Analysis of variance showed significant (*P* ≤ 0.05) differences for crop growth parameters under high temperature stress in canola (Table 1). All the growth-related parameters were decreased by high temperature stress while the intensity of reduction was varied in both genotypes. In 45S42 and Hiola-401, the interaction effect between TU supplementation × high temperature stress was significant for the dry weight while non-significant for plant height and root length. High temperature stress impaired the growth parameters as plant height, root length, and dry weight/plant decreased by 26.5, 21.2, and 34.7% in 45S42 and 27.4, 35.5, and 23.0% in Hiola-401respectively relative to control-no temperature stress, while more reduction was observed in control. However, TU applications effectively restored the growth in the canola plants (Table 1). Specifically, with TU supplementation, the plant height, dry weight plant^-1^, and root length were all improved by 21.2, 39.5, and 30.3% in 45S42 and 19.3, 33.8, and 27.0% in Hiola-401 as compared with control. Among TU applications, maximum improvement in plant growth parameters observed at TU_2_ (TU applied at anthesis stage) which improved plant height, dry weight plant^-1^, and root length by 25.8, 46.2, and 34.6% in 45S42 and 23.5, 41.5, and 30.0% in Hiola-401, respectively compared with TU_1_ (TU applied at seedling stage) with an improvement of 16.2, 32.8, and 26.1% in 45S42 and 15.1, 26.2, and 23.1% in Hiola-401 plant height, dry weight plant^-1^, and root length, respectively.

Analysis of variance showed a significant (*P* ≤ 0.05) effect of TU supplementations on plant water relations under high temperature stress in canola (Figs. 2 and 3). All the parameters were decreased by high temperature stress while the intensity of reduction was varied in both genotypes. In 45S42 and Hiola-401, the interaction effect between TU supplementation × high temperature stress was significant for the water potential and osmotic potential, while non-significant for turgor potential and leaf relative water content (LRWC). High temperature stress impaired the water relations as water potential, turgor potential, osmotic potential, and LRWC decreased under high temperature stress by 41.5, 31.3, 22.1, and 36.8% in 45S42 and 35.8, 33.3, 19.8, and 41.2% in Hiola-401, respectively, compared to control-no stress, while more reduction was observed in control. However, TU applications improved the plant water status in canola plants (Figs. 2 and 3). Nevertheless, TU applications improved the water potential, turgor potential, osmotic potential, and LRWC increased under high temperature stress by 20.4, 26.2, 12.4, and 14.7% in 45S42 and 17.9, 29.8, 10.7, and 13.4% in Hiola-401, respectively, compared with control. Among TU applications, the maximum improvement in water relations observed at TU_2_ which improved water potential, turgor potential, osmotic potential, and LRWC by 23.0, 31.8, 13.6, and 17.2% in 45S42 and 20.7, 38.4, 11.8, and 15.3% in Hiola-401, respectively compared with TU_1_ with an improvement of 17.7, 21.7, 10.9, and 12.8% in 45S42 and 15.0, 21.2, 9.60, and 11.6% in Hiola-401 in water potential, turgor potential, osmotic potential, and LRWC, respectively.

Results have revealed the significant (*P* ≤ 0.05) effect of TU supplementations on SPAD value under high temperature stress in canola, while the intensity of relation was varied in both genotypes (Table 1). In 45S42 and Hiola-401, the interaction effect between TU supplementation × high temperature stress was significant for the SPAD value. High temperature stress impaired the chlorophyll content index by reducing it up to 29.2% in 45S42 and 33.0% in Hiola-401, compared to the control, while more reduction was observed in the control. However, TU supplementation improved the foliar chlorophyll content (SPAD value) in canola plants (Table 1). Nevertheless, TU applications improved the SPAD value increased by 30.6% in 45S42 and 26.6% in Hiola-401, as compared with control under high temperature stress. Among TU applications, the maximum improvement in SPAD value was observed at TU_2_ which improved it by 33.4% in 45S42 and 30.5% in Hiola-401 compared with TU_1_) with an improvement of 26.7% in 45S42 and 22.8% in Hiola-401.

### Seed Yield and Yield quality parameters

Yield and yield-related attributes decreased significantly in canola, while the intensity of reduction varied depending on TU applications and growth conditions under high temperature stress (Table 1). In 45S42 and Hiola-401, the interaction effect between TU supplementation × high temperature stress was non-significant for yield attributes. High temperature stress reduced the yield parameters as 100-seed weight and seed yield plant^-1^ by 11.7 and 36.1% in 45S42 and 12.0 and 38.0% in Hiola-401, respectively relative to control-no temperature stress, while more reduction was observed in control-no TU applied. However, the TU-supplementation induced defense system improved the yield and yield attributes under high temperature stress (Table 1). Nevertheless, TU applications improved the 100-seed weight and seed yield plant^-1^ by 7.77 and 27.6% in 45S42 and 7.05 and 20.8% in Hiola-401, respectively, compared with control. Among TU applications, the maximum improvement in yield attributes at TU_2_ which improved 100-seed weight and seed yield plant^-1^ by 8.55 and 33.1% in 45S42 and 8.04 and 26.0% in Hiola-401, respectively compared with TU_1_ with an improvement of 6.98 and 22.3% in 45S42 and 6.06 and 15.6% in Hiola-401 in 100-seed weight and seed yield plant^-1^.

Under the stress of high temperatures, seed oil and protein levels were considerably impacted. Under high temperature stress in canola, seed oil content declined and seed protein increased dramatically, albeit the degree of reduction varied depending on TU treatments and growth circumstances (Table 3). In 45S42 and Hiola-401, the interaction effect between TU supplementation × high temperature stress was non-significant for yield attributes. Relative to control-no temperature stress, high temperature stress decreased the seed oil content by 32.4% in 45S42 and 34.7% in Hiola-401 and increased the seed protein by 31.9% in 45S42 and 20.5% in Hiola-401, whereas higher reduction was seen in control-no TU applied. However, the TU-supplementation-induced defense system increased the seed oil and decreased seed protein content under high temperature stress (Table 3). Nevertheless, TU applications improved the seed oil by 15.6% in 45S42 and 16.1% in Hiola-401 and decreased seed protein content by 20.3% in 45S42 and 16.0% in Hiola-401, respectively, compared with control. Among TU applications, maximum improvement in yield attributes at TU_2_ which increased the seed oil by 18.9% in 45S42 and 19.8% in Hiola-401 and decreased seed protein content by 24.3% in 45S42 and 20.6% in Hiola-401, respectively compared with TU_1_ with an increase of 12.3% in 45S42 and 12.5% in Hiola-401 in seed oil and seed protein content reduced by 12.5% in 45S42 and 11.3% in Hiola-401.

**Table 3:** Influence of supplemental thiourea applications on seed oil content, seed protein content, and fatty acid profile of two canola genotypes grown under high temperature stress.

Stress conditions (S)	Thiourea application (TU)	Seed oil content (%)		Seed protein content (%)		Palmitic acid (%)		Stearic acid (%)		Oleic acid (%)		Linoleic acid (%)		Linolenic acid (%)	
		G_1_	G_2_	G_1_	G_2_	G_1_	G_2_	G_1_	G_2_	G_1_	G_2_	G_1_	G_2_	G_1_	G_2_
NS	NA	33.3b	31.6c	17.4e	20.3d	4.63d	4.88e	2.58de	2.73e	33.0bc	31.4abc	18.6c	17.1c	13.1d	12.3c
	WA_1_	33.6b	32.0c	17.3e	20.3d	4.49d	4.84e	2.54de	2.68e	32.6c	29.6cd	18.8c	17.4c	13.7c	12.5c
	TU_1_	37.1a	35.0ab	20.4d	21.7c	3.97f.	4.28f.	2.22f.	2.39f.	28.4e	26.9f.	20.5b	18.7b	15.0b	14.7b
	WA_2_	34.0b	32.5c	17.7e	20.3d	4.33e	4.77e	2.51e	2.63e	32.4cd	27.2ef	19.1c	17.5c	13.9c	12.7c
	TU_2_	39.0a	37.3a	21.1cd	23.3c	3.41g	3.91g	1.98g	2.14g	26.9e	26.2f.	22.5a	19.9a	16.1a	15.3a
HT	NA	22.1d	20.3f.	22.8cd	23.6c	6.61a	6.87a	3.42a	3.56a	36.0a	32.9a	13.4f.	12.8e	9.44f.	8.84e
	WA_1_	22.3d	20.6f.	22.9c	23.7c	6.56a	6.82b	3.32b	3.46ab	35.4a	31.6ab	13.5ef	12.9e	9.76f.	8.92e
	TU_1_	25.2c	23.5de	26.4b	27.2b	5.95b	6.20c	2.96c	3.01c	32.2cd	29.7cd	14.7de	13.8d	11.8e	10.8d
	WA_2_	22.9d	21.0f.	23.1c	23.8c	6.50a	6.73b	3.25b	3.39b	34.5ab	29.8bcd	13.5ef	13.0e	9.91f.	9.10e
	TU_2_	27.0c	25.0d	28.9a	29.6a	5.49c	5.79d	2.61d	2.87d	31.0d	29.1de	15.3d	14.3d	12.2e	11.1d

LSD Least significant difference; Values sharing same case letter or without lettering, for a parameter, do not differ significantly (*P*** ≤ **0.05) by the LSD test.

*’**’***significant at *P* ≤ at 0.05, *P* ≤ at 0.01, *P* ≤ at 0.001 respectively.

NS no stress, HT High temperature stress, NA Control-no thiourea supplementation, WA_1_ = Water spray at seedling stage, TU_1_ = Thiourea supplementation at seedling stage, WA_2_ = Water spray at anthesis stage, TU_2_ = Thiourea supplementation at anthesis stage, G_1_ = 45S42, G_2_ = Hiola-40, ns = Non-significant

The fatty acid profile was negatively affected under high temperature stress in canola (Table 3). In 45S42 and Hiola-401, the interaction effect between TU supplementation × high temperature stress was non-significant for the fatty acid profile. High temperature stress reduced the yield and yield attributes as linolenic acid (LA) and linolenic acid (LLA) was reduced by 29.2 and 26.1% in 45S42 and 26.3 and 27.9% in Hiola-401, while, oleic acid (OA), stearic acid (SA), and palmitic acid (PA) was reduced by 10.2, 31.7, and 49.3% in 45S42 and 8.37, 29.6, and 51.8% in Hiola-401, respectively, relative to control-no temperature stress, while more reduction was observed in control-no TU applied. However, TU-supplementation induced defense system improved the LA and LLA, while, OA, SA, and PA were reduced under normal conditions and high temperature stress (Table 3). Nevertheless, TU applications improved the LA and LLA by 14.0 and 22.2% in 45S42 and 11.3 and 23.0% in Hiola-401, while, OA, SA, and PA were reduced by 14.1, 18.5, and 16.2% in 45S42 and 13.1, 14.1, and 17.2% in Hiola-401, respectively, compared with control. Among TU applications, the maximum improvement in yield attributes at TU_2_ which improved the LA and LLA by 18.2 and 25.3% in 45S42 and 14.1 and 25.3% in Hiola-401, while, OA, SA, and PA were reduced by 16.2, 18.5, and 14.1% in 45S42 and 14.1, 17.4, and 14.2% in Hiola-401, respectively compared with TU_1_ with an improvement of 9.96 and 19.1% in 45S42 and 8.47 and 20.7% in Hiola-401 in LA and LLA, while, OA, SA, and PA were reduced by 11.6, 13.6, and 12.1% in 45S42 and 12.0, 14.5, and 10.8% in Hiola-401, respectively.

### Biochemical parameters

Malondialdehyde content, H_2_O_2_ activity, and electrolyte leakage increased significantly (*P* ≤ 0.05) under high temperature stress causing oxidative stress; the exacerbation of high temperature stress led to more malondialdehyde production, H_2_O_2_ activity, and EL under severe stress (Table 2) that varied in canola genotypes. In 45S42 and Hiola-401, the interaction effect between TU supplementation × high temperature stress was malondialdehyde content and electrolyte leakage and non-significant for H_2_O_2_. High temperature stress triggered the activities of malondialdehyde content and H_2_O_2_ that led to increasing electrolyte leakage, compared to control-no stress, while more oxidative damage was observed in control-no TU applied. However, TU supplementation reduced oxidative stress by reducing lipid peroxidation under high temperature stress (Table 2). Nevertheless, TU applications reduced the malondialdehyde content, H_2_O_2_ activity, and electrolyte leakage by 19.08, 11.9, and 20.3% in 45S42 and 18.9, 10.2, and 18.1% in Hiola-401, respectively, compared with control. Among TU applications, maximum improvement in antioxidant enzymes at TU_2_ which improved malondialdehyde content, H_2_O_2_ activity, and electrolyte leakage by 23.7, 14.7, and 23.7% in 45S42 and 22.1, 12.4, and 21.2% in Hiola-401, respectively compared with TU_1_ with an improvement of 14.4, 9.08, and 17.0% in 45S42 and 15.8, 7.96, and 14.9% in Hiola-401 in malondialdehyde content, H_2_O_2_ activity, and electrolyte leakage.

**Figure 5:**
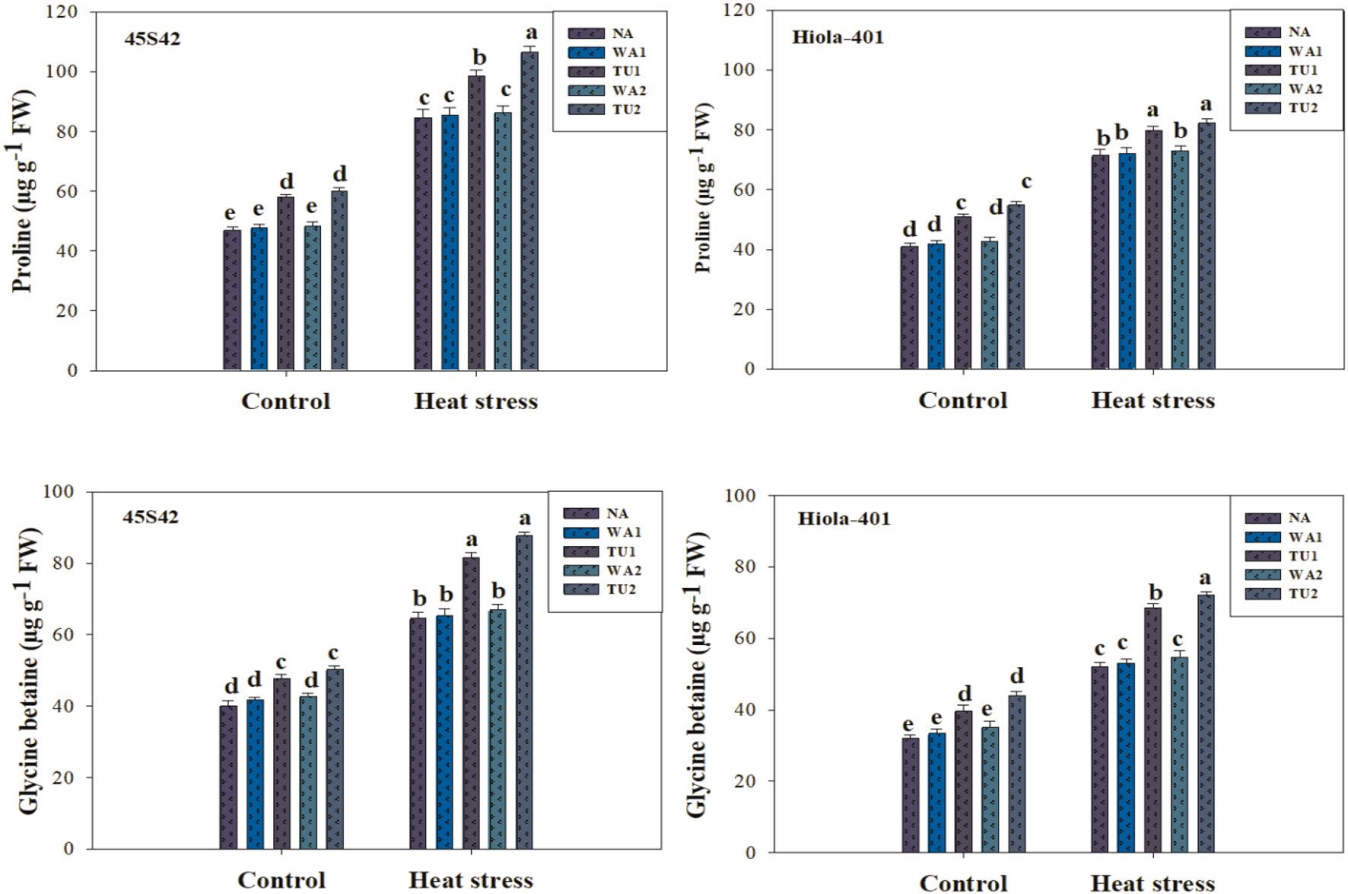
Effects of thiourea applications (NA = Control-no thiourea supplementation, WA_1_ = Water spray at seedling stage, TU_1_ = Thiourea supplementation at seedling stage, WA_2_ = Water spray at anthesis stage, TU_2_ = Thiourea supplementation at anthesis stage) on proline (μg g^−1^ FW) and glycine betaine (μg g^−1^ FW) content in canola genotypes under high temperature stress conditions.

Osmolyte and metabolite contents were significantly affected by TU supplementations under high temperature stress in canola, while it varied in both genotypes (Figs. 4 and 5). In 45S42 and Hiola-401, the interaction effect between TU supplementation × high temperature stress was significant for glycine betaine content and non-significant for proline content, total soluble proteins, and sugars. High temperature significantly improved the proline, glycine betaine, total soluble proteins, and total soluble sugars compared to the control. However, TU applications improved the proline, glycine betaine, total soluble proteins, and sugars in canola plants (Figs. 4 and 5). Nevertheless, TU applications improved proline, glycine betaine, total soluble proteins, and sugars increased by 22.7, 27.6, 13.3, and 14.9% in 45S42 and 19.2, 23.5, 12.8, and 11.6% in Hiola-401, compared with control. Among TU applications, maximum improvement in proline, glycine betaine, total soluble proteins, and sugars observed at TU_2_ which improved it by 26.5, 31.8, 15.6, and 16.9% in 45S42 and 12.1, 15.5, 22.2, and 28.2% in Hiola-401 compared with TU_1_ with an improvement of 18.9, 23.4, 11.0, and 11.6% in 45S42 and 13.5, 7.84, 16.2, and 22.7% in Hiola-401.

Analysis of variance showed a significant (*P* ≤ 0.05) effect of TU supplementations on antioxidant activities under high temperature stress in canola, while the intensity varied in both genotypes (Table 2). In 45S42 and Hiola-401, the interaction effect between TU supplementation × high temperature stress was non-significant for antioxidant enzymes. High temperature stress increased the antioxidant activities as catalase, peroxidase, and superoxide dismutase were increased by 57.8, 52.9, and 33.5% in 45S42 and 56.3, 52.1, and 29.0% in Hiola-401, respectively, compared to control-no stress, while more improvement was observed in TU applied plants. However, TU applications improved the activities of antioxidant enzymes in canola plants (Table 2). Nevertheless, TU applications improved the catalase, peroxidase, and superoxide dismutase increased under high temperature stress by 17.4, 26.5, and 22.5% in 45S42 and 21.8, 26.6, and 20.4% in Hiola-401, respectively, compared with control. Among TU applications, the maximum improvement in antioxidant enzymes at TU_2_ which improved catalase, peroxidase, and superoxide dismutase by 20.6, 29.3, and 23.6% in 45S42 and Hiola-401, respectively compared with TU_1_.

### Relationships between measured traits

**Figure 6:**
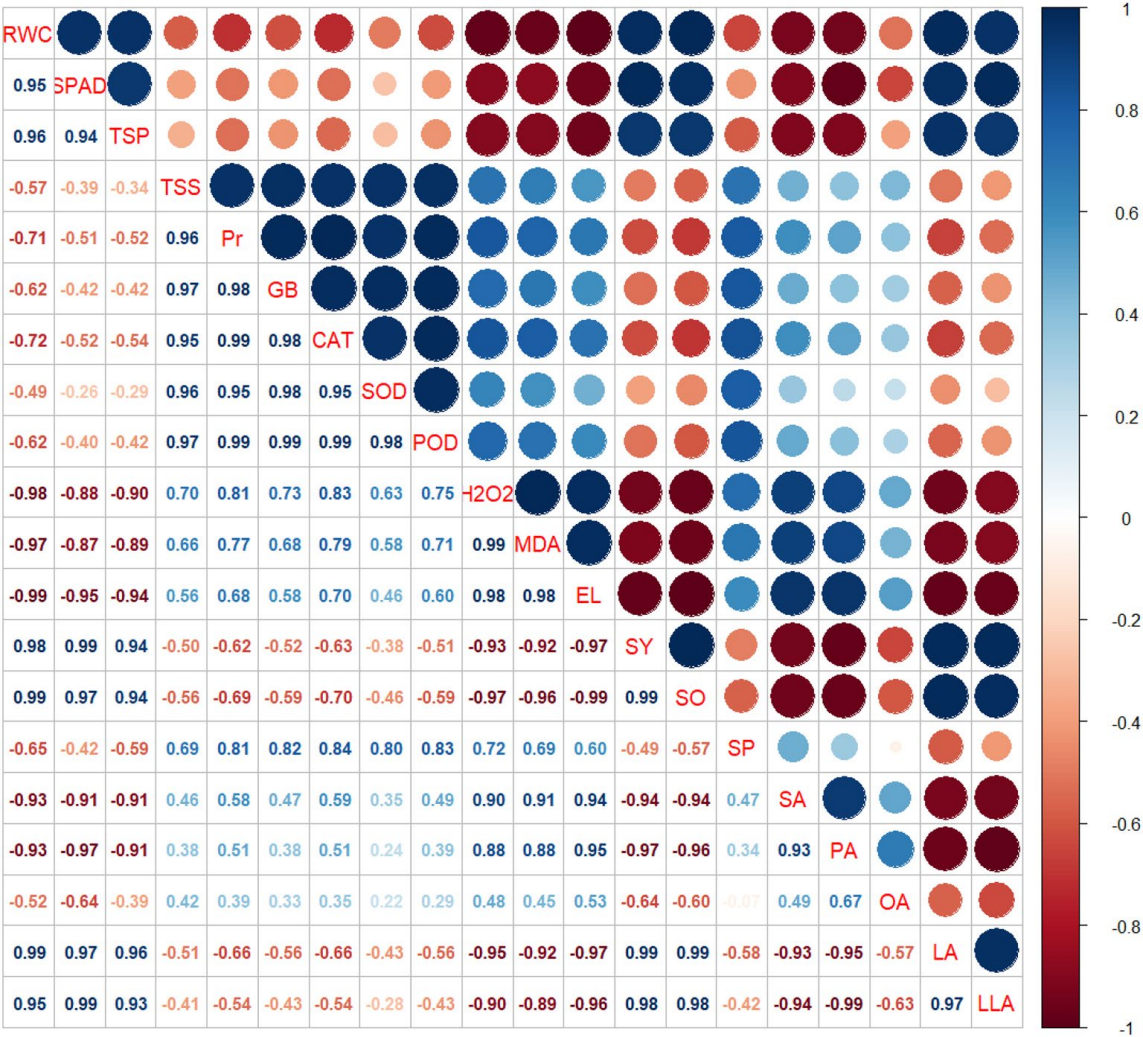
Pearson correlation of canola growth and development, physiological, biochemical, yield, and yield quality parameters under thiourea supplementation under normal and high temperature stress in canola. RWC (relative water content), SPAD (the soil plant analysis development for foliar chlorophyll), TSP (total soluble proteins), TSS (total soluble sugars), Pr (proline), GB (glycine betaine), CAT (catalase), SOD (superoxide dismutase), POD (peroxidase), H_2_O_2_ (hydrogen peroxide), MDA (malondialdehyde), EL (electrolyte leakage), SY (seed yield), SO (seed oil), SP (seed protein), SA (stearic acid), PA (palmitic acid), OA (oleic acid), LA (linoleic acid), LLA (linolenic acid).

**Figure 7:**
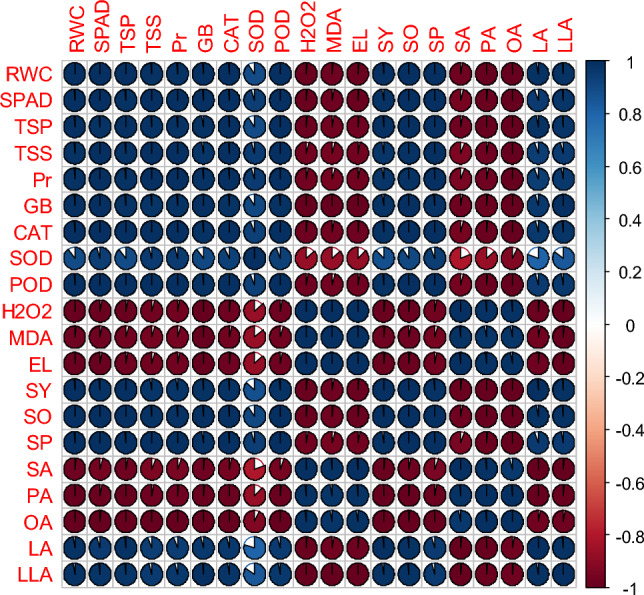
Pearson correlation of plant growth and development, physiological, biochemical, yield, and yield quality parameters under thiourea supplementation under normal in canola genotype-45S42. RWC (relative water content), SPAD (the soil plant analysis development for foliar chlorophyll), TSP (total soluble proteins), TSS (total soluble sugars), Pr (proline), GB (glycine betaine), CAT (Catalase), SOD (superoxide dismutase), POD (peroxidase), H_2_O_2_ (hydrogen peroxide), MDA (malondialdehyde), EL (electrolyte leakage), SY (seed yield), SO (seed oil), SP (seed protein), SA (stearic acid), PA (palmitic acid), OA (oleic acid), LA (linoleic acid), LLA (linolenic acid).

**Figure 8:**
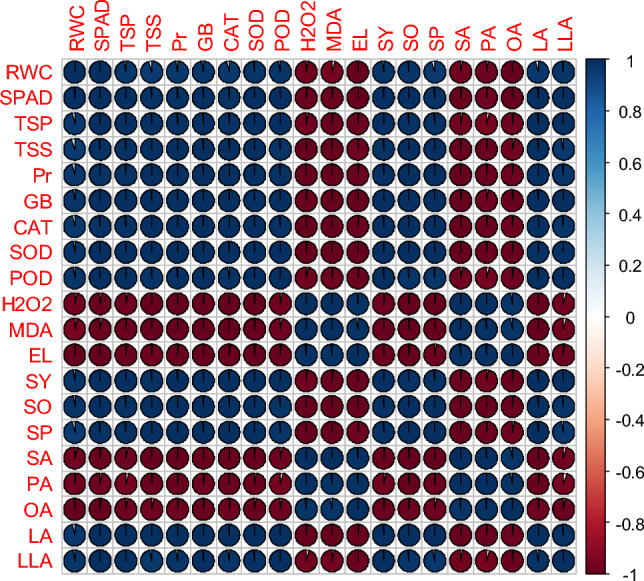
Pearson correlation of plant growth and development, physiological, biochemical, yield, and yield quality parameters under thiourea supplementation under high temperature stress in canola genotype-45S42. RWC (relative water content), SPAD (the soil plant analysis development for foliar chlorophyll), TSP (total soluble proteins), TSS (total soluble sugars), Pr (proline), GB (glycine betaine), CAT (catalase), SOD (superoxide dismutase), POD (peroxidase), H_2_O_2_ (hydrogen peroxide), MDA (malondialdehyde), EL (electrolyte leakage), SY (seed yield), SO (seed oil), SP (seed protein), SA (stearic acid), PA (palmitic acid), OA (oleic acid), LA (linoleic acid), LLA (linolenic acid).

**Figure 9:**
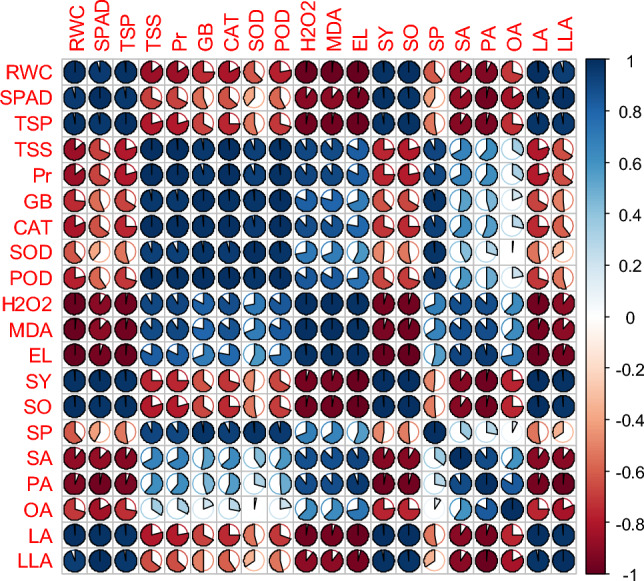
Pearson correlation of plant growth and development, physiological, biochemical, yield, and yield quality parameters under thiourea supplementation under normal in canola genotype- Hiola-401. RWC (relative water content), SPAD (the soil plant analysis development for foliar chlorophyll), TSP (total soluble proteins), TSS (total soluble sugars), Pr (proline), GB (glycine betaine), CAT (catalase), SOD (superoxide dismutase), POD (peroxidase), H_2_O_2_ (hydrogen peroxide), MDA (malondialdehyde), EL (electrolyte leakage), SY (seed yield), SO (seed oil), SP (seed protein), SA (stearic acid), PA (palmitic acid), OA (oleic acid), LA (linoleic acid), LLA (linolenic acid).

**Figure 10:**
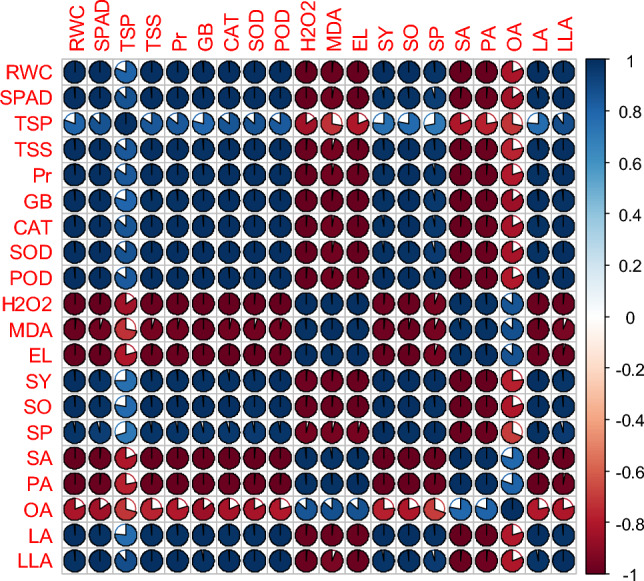
Pearson correlation of plant growth and development, physiological, biochemical, yield, and yield quality parameters under thiourea supplementation under under high temperature stress in canola genotype-Hiola-401. RWC (relative water content), SPAD (the soil plant analysis development for foliar chlorophyll), TSP (total soluble proteins), TSS (total soluble sugars), Pr (proline), GB (glycine betaine), CAT (catalase), SOD (superoxide dismutase), POD (peroxidase), H_2_O_2_ (hydrogen peroxide), MDA (malondialdehyde), EL (electrolyte leakage), SY (seed yield), SO (seed oil), SP (seed protein), SA (stearic acid), PA (palmitic acid), OA (oleic acid), LA (linoleic acid), LLA (linolenic acid).

The significant and positive correlation between catalase, peroxidase, and superoxide dismutase, as well as leaf proline and glycine betaine content under high temperature stress, while all the growth and yield, and water relations had a significant negative correlation with malondialdehyde production, H_2_O_2_ activity, and EL in both genotypes (Figs. 6–10). A significant and positive correlation was noted between plant growth and water relations. Interestingly, seed yield and seed quality parameters had shown a highly positive correlation with the activity of antioxidant enzymes, soluble sugars and proteins, and osmolyte activities in both genotypes. The correlation between saturated and unsaturated fatty acids was negative. Nonetheless, a significant and negative correlation was detected between malondialdehyde production, H_2_O_2_ activity, EL and water relations, growth, and yield attributes in both genotypes (Figs. 6–10).

### Clustered heatmap

**Figure 11:**
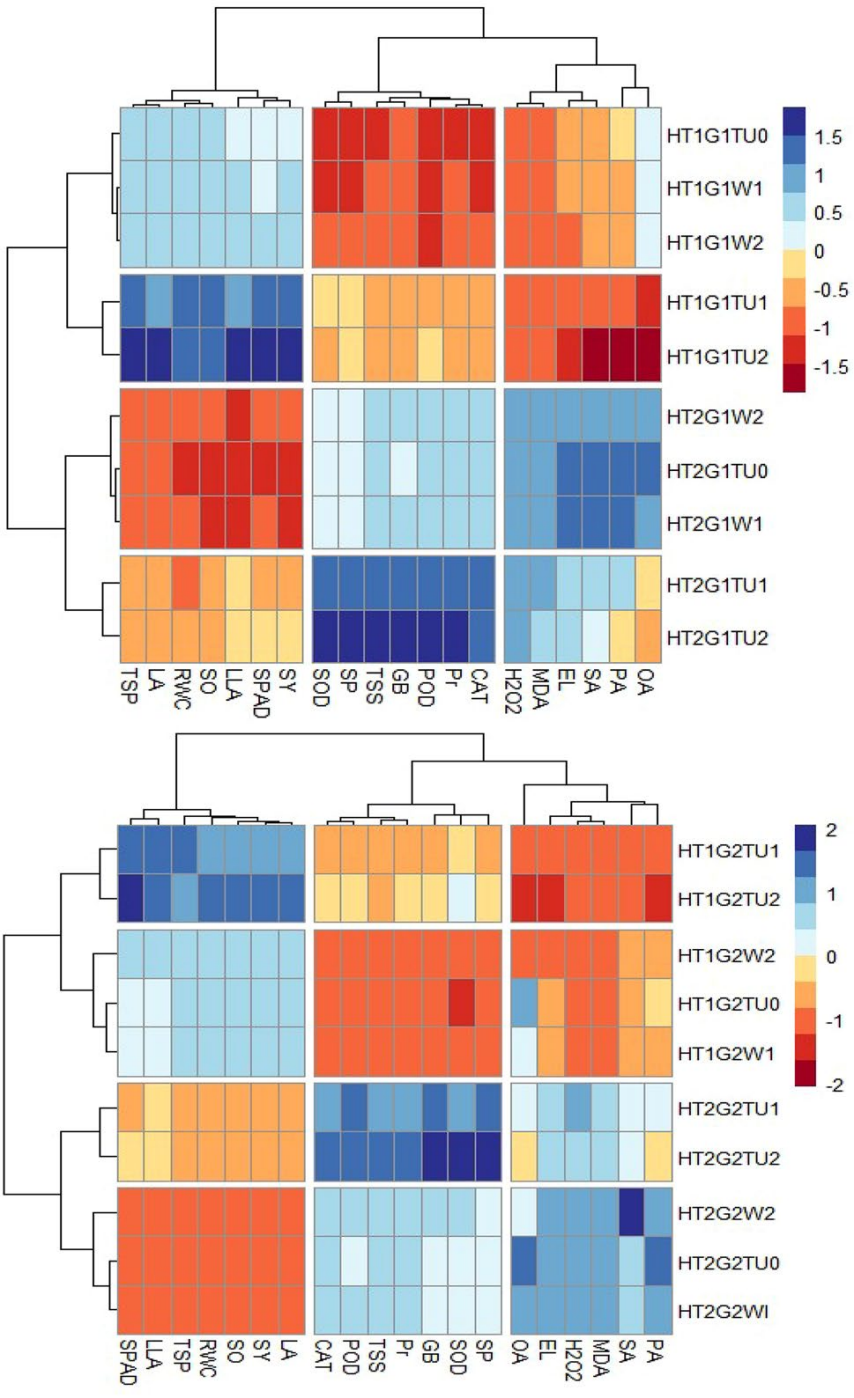
Clustered heatmap of a number of physiological, biochemical, and quality attributes, including RWC (relative water content), SO (Seed oil), TSP (Total soluble proteins), LA (Linoleic acid), SPAD (The Soil Plant Analysis Development for foliar chlorophyll), SY (Seed yield), LLA (Linolenic acid), TSS (Total soluble sugars), GB (Glycine betaine), POD (Peroxidase), Pr (Proline), CAT (Catalase), SOD (Superoxide dismutase), SP (Seed protein), H_2_O_2_ (hydrogen peroxide), MDA (Malondialdehyde), EL (Electrolyte leakage), SA (Stearic acid), PA (Palmitic acid), OA (Oleic acid) of canola genotype (G_1_ = Hiola-401 and G_2_ = 45S42) grown under normal (no stress) and high temperature stress (35°C), WA_1_ = Water spray at seedling stage, TU_1_ = Thiourea supplementation at seedling stage, WA_2_ = Water spray at anthesis stage, TU_2_ = Thiourea supplementation at anthesis stage. The rows are representing different parameters and each column represents different treatments.

**Figure 12:**
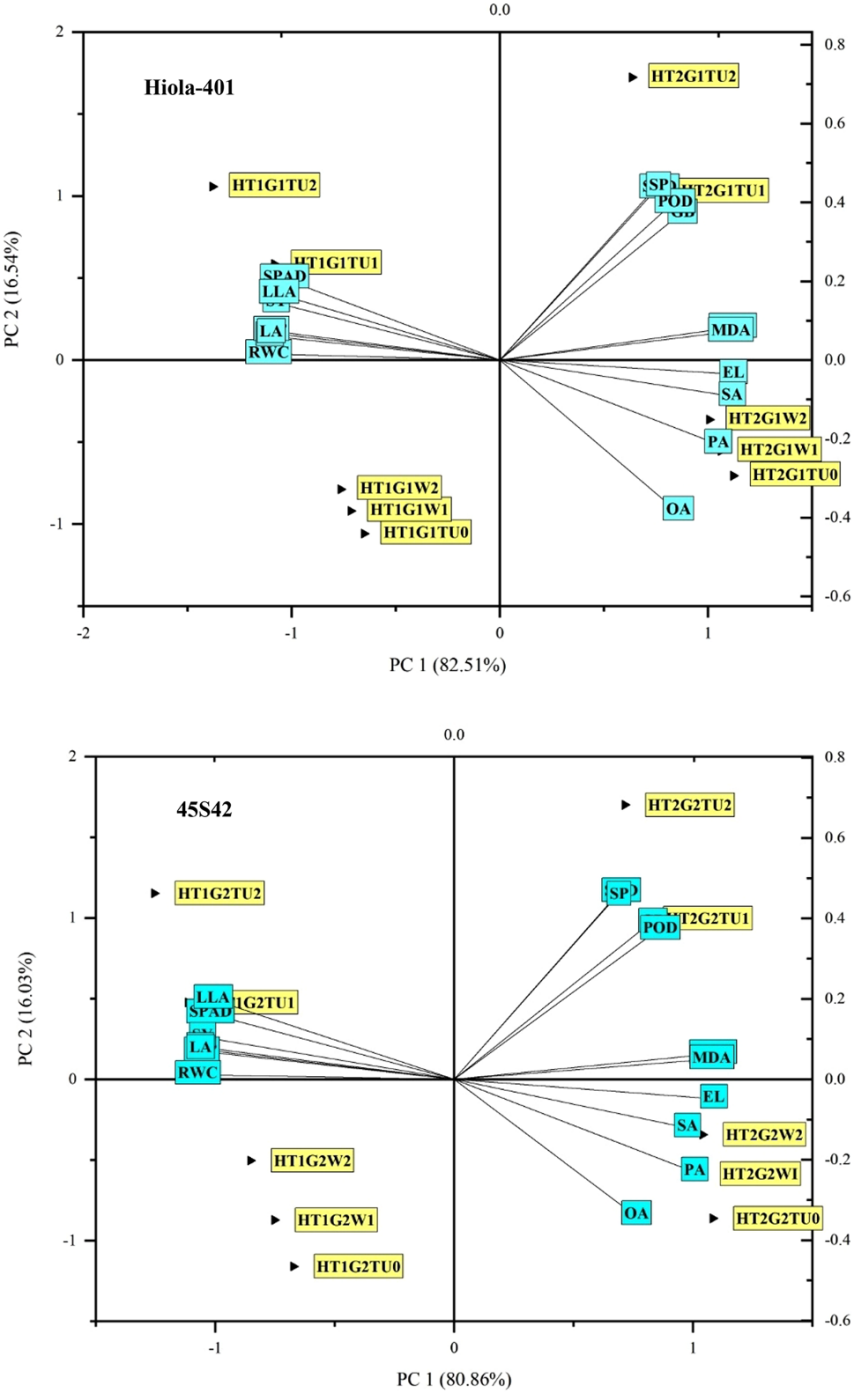
Principal component analysis (PCA) of a number of physiological, biochemical, and quality attributes, including RWC (relative water content), SO (Seed oil), TSP (Total soluble proteins), LA (Linoleic acid), SPAD (The Soil Plant Analysis Development for foliar chlorophyll), SY (Seed yield), LLA (Linolenic acid), TSS (Total soluble sugars), GB (Glycine betaine), POD (Peroxidase), Pr (Proline), CAT (Catalase), SOD (Superoxide dismutase), SP (Seed protein), H_2_O_2_ (hydrogen peroxide), MDA (Malondialdehyde), EL (Electrolyte leakage), SA (Stearic acid), PA (Palmitic acid), OA (Oleic acid) of canola genotype (G_1_ = Hiola-401 and G_2_ = 45S42) grown under normal (no stress) and high temperature stress (35 °C), WA_1_ = Water spray at seedling stage, TU_1_ = Thiourea supplementation at seedling stage, WA_2_ = Water spray at anthesis stage, TU_2_ = Thiourea supplementation at anthesis stage.

In the heatmap, different colors correspond with the relative abundance of the selected variables (physiological, biochemical, yield, and yield quality) and the variations of these variables in various states during high-temperature stress. Similar values of the parameter are shown by the same color. Each parameter's value is expressed as a proportion of the related control. Both genotypes have shown that seed yield and yield quality parameters were directly clustered with biochemical attributes showing the relationship and dependency of seed yield and seed oil content (Figs. 11 and 12). Seed yield, seed oil content, SPAD value, and soluble sugars clustered in the same group. Biochemical attributes including stressed indicators and antioxidants were directly and indirectly clustered with seed quality parameters including oil content and fatty acid profile showing more differences among canola genotypes under heat stress.

### Principal component analysis (PCA)

The principal component analysis was carried out to find the role of thiourea on canola genotypes Hiola-401 and 45S42 under heat stress conditions (Fig. 12). In genotype Hiola-401, the PCA showed 82.5% variability by PC1 and 16.5% by PC2 axis. Maximum attributes of genotype G1 = Hiola-401 were plotted in HT2 = high temperature stress (35/25 °C day/night, TU_1_ = foliage TU supplementation at seedling stage (Principal growth stage 3: Stem elongation, BBCH Scale-39) to show a close relationship of parameters in this genotype under heat stress conditions. The MDA, H_2_O_2_, SP = Seed protein, OA = Oleic acid, POD, EL = electrolytic leakage, and SA = Stearic acid in canola were shown towards HT_2_, while SPAD, linolenic acid, RWC contents were plotted NS = control-no stress (25/18 °C day/night) showing that G1 = Hiola-401 are sensitive to heat stress they reduce the seed protein and fatty acid concentration under HT2 = HT2 = high temperature stress (35/25 °C day/night) (Fig. 12).

In the genotype, the PCA showed 80.9% variability by PC1 and 16.03% by PC2 axis. Maximum attributes of genotype G2 = 45S42 were plotted in HT2 = high temperature stress (35/25 °C day/night, TU_1_ = foliage TU supplementation at seedling stage (Principal growth stage 3: Stem elongation, BBCH Scale-39) to show a close relationship of parameters in this genotype under heat stress conditions (Fig. 12). The MDA, H_2_O_2_, SP = Seed protein, OA = Oleic acid, POD, EL = electrolytic leakage, and SA = Stearic acid in canola were shown towards HT_2_ and WA_1_ = water spray at seedling stage (Principal growth stage 3: Stem elongation, BBCH Scale-39), while SPAD values, linolenic acid, linoleic acid, RWC, and TSS contents were plotted NS = control-no stress (25/18 °C day/night) showing that application of TU_1_ supported the plant growth by improving its chlorophyll contents and total soluble sugar under heat stress (Fig. 12).

## Discussion

Under the umbrella of the climate change scenario, high temperature stress lowered the performance of canola plants, reducing the yield and yield quality parameters^13^,^18^. To date, the studies have exhibited the stage sensitivity of high temperature stress on canola seed yield but the role of TU to regulate seed oil quality needs to be studied under normal and unfavorable conditions. The present study has evaluated the role of TU as a redox modulator to alleviate heat stress damages and as a growth regulator to improve yield and yield quality parameters including fatty acid profile. The results showed the acceptance of the hypothesis that TU supplementation alleviated the oxidative stress caused by high temperature stress and improve the seed oil quality in canola (Tables 1–2; Figs. 1–5).

High temperature is often coupled with drought stress to affect plant growth and development^2,33^. Results have shown that high temperature stress negatively affected the growth parameters in canola genotypes and maximum reduction was observed in Hiola-401 that might be due to to the reduction in foliar chlorophyll content, gas exchange and photosynthetic efficiency. A significant decrease in growth attributes was observed under heat treatment which might be due to the damage to the photosynthetic machinery that reduces the carbon fixation^62^. However, there was a considerable increase in plant height, root length, and biomass accumulation in canola genotypes under the TU-supplementation as compared to the control (Table 1) and 45S42 showed maximum improvement in growth attributes as compared with Hiola-401. Thus, the observed phenotype demonstrated the effect of TU to alleviate heat-induced damages which is also corroborated with the findings of Khanna et al.^63^ and Srivastava et al.^64^ who stated that TU application can improve plant growth and development due to the improvement in chloroplast production and photosynthetic rate. The improvement in root length is a key adaptive feature of high temperature stress tolerance because proper growth and development were dependent upon active root growth^63,65^ which was significantly increased due to TU supplementation^66^.

The decrease in growth was brought on by the negative effects of high temperature perturbation on plant water relations, as water potential, turgor potential, relative water content, and foliar chlorophyll (SPAD values) (Figs. 1 and 3; Table 1). The reduction in plant water status affected growth as the reduction in turgor pressure reduces the cell elongation that affects the plant height and biomass accumulation^1,33^. High temperature stress generally increases the plant evapotranspiration rate to cool the shoot system; inevitably lowering the tissue water status and osmotic adjustments in different species; hastening leaf senescence owing to lower chlorophyll content and photosynthetic activity^57,67, 68, 71^. Thiourea-supplemented plants exposed to high temperature stress exhibited a significant improvement of water relations including the relative water content, turgor pressure, and SPAD values, while more improvement was observed in 45S42 as compared with Hiola-401 (Figs. 1 and 3; Table 1). The results highlighted the positive role of TU in maintaining plant water status, predominantly in turgor maintenance that sustained the gaseous exchange activity to support plant growth^35,69^.

High temperatures stress reduced the seed yield parameters and seed quality traits^65^. The findings of this study demonstrated that high temperature stress decreased seed oil content and quality, with canola genotypes showing a rise in saturated fatty acids and a decrease in unsaturated fatty acids (Table 3). However, an increase in seed protein content was also observed in canola genotypes under high temperature stress due possibly to the dilution effect. High temperature negatively affected plant metabolism and especially gaseous exchange, favouring the accumulation of proteins over the seed oil content^70,71^. High temperature stress at sensitive crop stage reduced the activities of starch-metabolizing enzymes like soluble starch synthase, ADP-glucose pyrophosphorylase, and starch-branching enzyme, which limited starch synthesis^72^. Pokharel et al.^72^ reported that high temperature stress increased the stearic acid and palmitic content and decreased the linoleic acid and linolenic acid contents in canola. Results revealed that TU applications improved the seed oil content and seed quality, regulating the unsaturated fatty acids, while, saturated fatty acids were decreased in canola genotypes. Unsaturated fatty acids, such as linoleic acid and linolenic acid were increased when TU was applied at the reproductive stage as compared to the vegetative stage. Photo-assimilate production was increased at the sink due to TU applications that synchronized with acetyl-CoA carboxylase to improve the synthesis linked with the usage of stored carbohydrates for oil synthesis^73,74^. These conditions could all result in higher levels of metabolites and increased activity of the higher enzyme (phosphoenolpyruvate carboxylase), which would indicate that plants receiving TU supplements had efficient silicle photosynthesis^75^. The role of TU in improving fatty acid profile lies in the fact that TU molecule constitutes nitrogen and sulfur that may contribute to improving the stability of cell membrane due to the improvement in fatty acid profile^76^ as the essential components of biological membranes are fatty acids. The responses of the two canola genotypes were comparable with regards to fatty acid composition but the intensity of response was different due to different genetic potential. Previous research demonstrated the importance of S and N in enhancing oil quality, as Jiang et al.^77^ discovered a substantial relationship between N and S that helped the plants to improve unsaturated fatty acid by reducing saturated fatty acid contents. Supplementing with sulfhydryl TU increased the production of acetyl co-enzyme A, a precursor to long-chain fatty acids, increasing seed oil content and, eventually, oil recovery and fatty acid profile^74,78^.

Compatible osmolytes/metabolites are well known for their role in a wide range of functions, i.e., protection of stress enzymes, maintenance of biological membrane, working as osmoticum to maintain cell turgor, and detoxifying roles against oxygen radicals in plants^35,62, 79^. Among the two stages of TU foliar spray, TU supplementation at anthesis was more effective in improving plant growth and development under high temperature stress. Thiourea applied at the seedling stage may be utilized for the development of vegetative growth while TU applications at anthesis, which was counteracted by high temperature may utilize the available TU for the activation of plant defense system against heat stress. Heat stress strongly affected the ROS scavenging antioxidative defense mechanism enzymes, correlated with heat stress tolerance, and defined as the characteristic of heat stress tolerance (Figs. 6–8). Results showed that ROS scavenging enzymes, compatible osmolytes/metabolites including catalase, superoxide dismutase, peroxidase, total soluble proteins/sugars, proline, and glycine betaine significantly increased in canola genotypes under high temperature stress, while more increase was examined in 45S42 as compared with Hiola-401 (Table 2; Figs. 4 and 5). Accrual of proline and glycine betaine, in TU-treated seedlings improved the tolerance against heat stress and improves redox potential to augment cellular osmotic adjustments under stress conditions^42^. Soluble sugars, proteins, and total soluble significantly improved the plant water which led to improved plant growth and development. The role of plant metabolites and osmolyte production led to improved crop growth and development under high temperature stress, perhaps, by improving carbon fixation and stomatal regulations^80^. The trends for other antioxidants were corroborated with superoxide dismutase by TU applications under high temperature stress (Figs. 4 and 5).

Heat stress suppressed canola growth, dry matter accumulation, water relation, and seed yield in two genotypes tested. Specifically, the high temperature stress increased the lipid peroxidation and over-accumulation of reactive oxygen species, and the latter is believed to be involved in disturbing the membrane stability as it increased the permeability of biological membrane in both genotypes due to the efflux of cell contents and ions (Tables 1 and 2; Figs. 6–8). Plant growth and development may be constrained as a result of the ionic imbalance and membrane permeability disruption^81^. Interestingly, TU supplementation played an important role in detoxifying ionic toxicity and improving the nutrient contents while concomitantly reducing the accumulation of H_2_O_2_, malondialdehyde, and cell membrane permeability^82,83^. Similar results to ours have been reported in the literature, where high temperature stress led to a considerable rise in H_2_O_2_ and MDA (Table 2), and TU supplementation, particularly at anthesis, was effective in mitigating the adverse effect of high temperature stress on canola genotypes.

Superoxide dismutase is the first line of defense against stress conditions that convert the radicle species, i.e., O_2_^·^ into O_2_ and H_2_O_2_, which needs to be further detoxified by the catalase to H_2_O and O_2_^81^ The present results indicated that TU induced defense system significantly improved heat stress tolerance in canola. Catalase activity increased with TU supplementation while the variations were observed among the genotypes, the reason could be the improvement in the gene expression that induced the catalase activity as defined by Srivastava et al.^84^ High catalase activity is associated with enhanced scavenging of H_2_O_2_ as suggested by Foyer et al.^85^ that would be primarily carried out by TU induced catalase activity in canola genotypes. These findings imply that TU treatment may have enhanced redox-mediated stress tolerance. Thiourea supplementation at both growth stages caused a noticeable increase in the activity of superoxide dismutase, while TU supplementation at anthesis was more effective, indicating the higher efficacy of the antioxidant enzyme. At the plant level, the production of ROS scavenging/producing enzymes and antioxidant metabolites regulate the redox state^86,87^.

Heat stress at anthesis adversely affected the sensitive process of yield attribute formation in canola genotypes. The reduction in growth attributes including plant height, root length, and plant water relations leading to lower seed yield and related attributes in canola genotypes (Table 1; Figs. 2 and 3). High temperature stress might hasten the grain filling processes, thus reducing the duration of grain filling. It is generally believed that the reduction in grain filling duration under elevated temperature may not fully compensate for the grain filling rate, because the limited supply of photo-assimilates may limit grain filling rate^1,33, 88^. On the other hand, TU supplementation restored crop yield by ameliorating the adverse effects of oxidative stress by the antioxidant defense system in canola genotypes. Maximum improvement in seed yield/plant was observed in 45S42 compared with susceptible one Hiola-401. The improvement in plant growth and physiological parameters due to the upregulation of the plant defense system and improved source-sink relation led to improved seed yield and yield quality in oilseed crops^28,71^. Under high temperature stress, TU application upregulated the plant hydration status to an optimal level by regulating the osmotic potential to sustain water uptake and maintain the LRWC which led to improved crop growth and yield^89^.

The whole experiment was performed in the growth rooms under controlled conditions to ensure internal temperature, and many other factors including water and nutrients, etc, were reproducible. Taken together, the results accepted the proposed hypothesis and confirmed the important role of TU application to improve seed oil and oil quality, including unsaturated fatty acid content in canola genotypes under normal and high temperature stress. Thus, the positive impact of TU on crop performance under control and heat stress conditions greatly increases its versatility to be applied under a realistic field scenario.

## Conclusions

The current study demonstrated that high temperature stress reduced canola growth, plant water status, seed yield, and yield quality in two genotypes; with more reduction in Hiola-401 compared with 45S42. High temperature stress triggered lipid peroxidation (malondialdehyde), accumulation of hydrogen peroxide, and electrolyte leakage, apparently by desynchronizing the mechanism of ROS-detoxification. Interestingly, TU supplementation restored high temperature-induced inhibitory effects on the canola development, physiology, seed yield, and seed oil content and seed oil quality; these favorable observations might primarily be attributed to lesser water loss, strengthened chlorophyll index, consistent leaf turgor, and greater availability of scavenging ROS amount. In addition, TU applications at anthesis improved the canola seed oil content and seed oil quality by increasing the amount of unsaturated fatty acids in two genotypes. Moving forward, further investigations are required to examine how HSPs and other heat shock factors interact under TU applications to confer heat stress tolerance to canola and other species.

## Data Availability

All data generated or analyzed during this study are included in this published article.
